# Integrated Transcriptomic and Targeted Metabolomic Analysis Reveals the Key Genes Involved in Triterpenoid Biosynthesis of *Ganoderma lucidum*

**DOI:** 10.3390/jof11010057

**Published:** 2025-01-13

**Authors:** Xiaolan Xu, Chunxia Li, Fangjing Wu, Shuangshuang Zhao, Tiqiang Chen, Haihong You, Yijie Lin, Xiaoxing Zou

**Affiliations:** 1 College of Bee Science and Biomedicine, Fujian Agriculture and Forestry University, Fuzhou 350002, China; xlxufz@126.com (X.X.); 18887266780@139.com (C.L.); 13088247367@163.com (F.W.); 18638694709@163.com (S.Z.); 18150852107@163.com (H.Y.); 13859218293@163.com (Y.L.); 2 Institute of Edible & Medicinal Mushroom, Fujian Academy of Agriculture Sciences, Fuzhou 350014, China; 3 Forestry College, Fujian Agriculture and Forestry University, Fuzhou 350002, China

**Keywords:** *Ganoderma lucidum*, ganoderic triterpenoid, transcriptomic analysis, targeted metabolomic analysis, transcription factors, correlation analysis

## Abstract

*Ganoderma lucidum* is a traditional Chinese medicinal fungus, and ganoderma triterpenoids (GTs) are one of the main bioactive compounds. These compounds have various pharmacological functions, including anti-tumor, antioxidant, anti-inflammatory, liver-protective, and immune-regulating effects. However, the manner in which they accumulate, and their biosynthesis mechanisms remain unclear. To screen for the genes that are involved in the biosynthetic pathway of GTs, this study analyzed the differential metabolites and differentially expressed genes (DEGs) among different growth stages of *G. lucidum*, including the primordia (P), the matured fruiting body (FM), and the post-spore fruiting bodies (FP) using targeted metabolomics and transcriptomics analysis, respectively. The results showed that a total of 699 components were detected, including lignans, terpenoids, amino acids and derivatives, and phenolic acids, among others. Among them, a total of 112 types of triterpenes were detected. Compared with the primordia, there were eight differential metabolites of triterpenoids, with three decreasing and five increasing in the FM stage. A comparison between the FM stage and the FP stage revealed that there were 13 differential metabolites of triterpenoids. A transcriptomics analysis showed that there were 371 DEGs in the P_vs_FM group, including 171 down-regulated genes and 200 up-regulated genes. In the FM_vs_FP group, 2567 DEGs were identified, with 1278 down-regulated genes and 1289 up-regulated genes. Through targeted metabolomics and transcriptome correlation analysis, six TFs and two CYP450s were significantly associated with four triterpenoid components. The results showed that these TFs and CYP450s were positively or negatively correlated with the four triterpenoid components. In addition, interestingly, some flavonoids and phenolic compounds, which have been reported in plants, were also detected in *G. lucidum*, indicating that it has the potential to be engineered into a strain capable of synthesizing flavonoid compounds. This study provides useful information about key genes involved in GT biosynthesis, but further exploration and in-depth research are needed to better understand the functions of these genes.

## 1. Introduction

*Ganoderma lucidum*, known as lingzhi in China, is a traditional Chinese medicinal fungus with a complex chemical composition [1]. Over 400 compounds have been identified in *G. lucidum*, including triterpenoids, polysaccharides, nucleosides, furan derivatives, steroids, alkaloids, and peptide amino acids, among others [2]. Among them, ganoderma triterpenoids (GTs) and polysaccharides are the most important pharmacologically active ingredients [2]. GTs have complex structures and are classified into different types, such as ganoderic acids, lucidenic acids, ganoderols, ganoderals, and ganoderones, based on their functional groups and side chains. Numerous studies have shown that GTs possess various pharmacological characteristics, including immune modulation, anti-tumor, hepatoprotection, detoxification, and anti-fatigue properties [3,4,5].

Significant progress has been made in the chemical and pharmacological research of *G. lucidum*, but its biological studies are still limited, and the biosynthetic pathway of triterpenoids has not yet been fully elucidated. The biosynthetic pathway of GTs is through the mevalonate pathway to generate lanosterol, which is then converted into various structures of GTs, such as ganoderic acid, ganoderiol, and lucidenic acid, under the action of glycosyltransferases and cytochrome P450 (CYP450) enzymes [6]. The CYP450s are monooxygenase catalytic proteins and constitute a diverse superfamily involved in the biosynthesis of terpenoids, alkaloids, flavonoids, fatty acids, and other compounds in plants. In the genome of *G. lucidum*, a total of 219 CYP450 genes have been annotated, and 21 have been characterized [6]. The function of *CYP5150L8*, which positively correlated with lanosterol synthase, in catalyzing triterpenoid biosynthesis was verified [7]. Subsequently, based on this research, 158 CYP450s were screened using *Saccharomyces cerevisiae* as the host, and several CYP450s capable of producing type II ganoderic acid were identified. Moreover, the pathway and reaction mechanism of CYP512W2 catalyzing the generation of type II ganoderic acid from type I ganoderic acid were elucidated. Therefore, constructing engineered yeast strains to produce type II ganoderic acids by screening CYP450 enzymes would be a potential commercial approach [8].

In addition, the synthesis of GTs is regulated positively or negatively by transcription factors (TFs). TFs, also known as trans-acting molecules, could regulate the transcription level of target genes by binding to cis-acting elements in the promoter region to modulate the content of secondary metabolites. TFs play a crucial role in the synthesis and regulation of pharmacologically active components in medicinal plants, such as terpenoids, flavonoids, alkaloids, and other secondary metabolites [9]. After the *G. lucidum* genome was sequenced, about 600 transcription factors were annotated, including families such as CCHC, C2H2, Zn2Cys6, GCN5, PHD, SET, and HDAC [6]. So far, some transcription factor functions have been validated. Silencing *GCN4*, *GlSwi6*, and *Skn7* resulted in changes in the content of GTs that were consistent with changes in the ROS content, suggesting these genes regulate GTs synthesis through ROS signaling [10,11,12]. Silencing *PacC*, *MADS1* and *CRZ1* leads to increases or decreases in the content of GTs, indicating these genes were also involved in the regulation of GTs [13,14]. Additionally, the transcription factor *GCN4* can directly bind to the *GlMPC* promoter region, activating *GlMPC* expression and participating in the regulation of secondary metabolism under low nitrogen conditions [15]. The MeJA-responsive transcription factor *Glmhr* can influence lanosterol synthesis and accumulation by regulating the expression of *FPS* [16]. TFs play a crucial regulatory role in the metabolism and synthesis of triterpenoids in *G. lucidum*, but most of them are involved in triterpene biosynthesis and growth processes remain unstudied.

The growth of *G. lucidum* goes through several stages, including the primordium, fruiting body formation, fruiting body maturation, spore maturation, and post-spore harvest. We used UPLC-QTOF/MS technology to detect a total of 89 chemical compounds at different growth stages of *G. lucidum*, among which one triterpenoid compound was found in the mycelia, 45 triterpenoid compounds were found in the primordia, and 48 triterpenoid compounds were found in the matured fruiting body [16]. The determination of the chemical components of *G. lucidum* has been reported; however, there are no reports on using targeted metabolomics to determine differences in components among the primordia (P), the matured fruiting body (FM), and the post-spore fruiting body (FP) of *G. lucidum*. To gain a deeper understanding of the compositional differences at different stages of *G. lucidum* and to screen genes related to GTs synthesis, this study analyzed the primordia, mature fruiting bodies, and post-spore fruiting bodies using metabolomics and transcriptomics. This study provided additional data for the study of the biosynthetic pathway of GTs.

## 2. Materials and Methods

### 2.1. Samples

**Figure 1:**
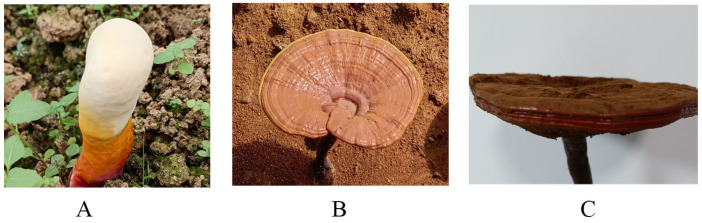
The samples of *G. lucidum* used for this study. (**A**) Primordia (P); (**B**) Matured fruiting body (FM); (**C**) the fruiting body after spore production (FP).

The samples of *G. lucidum* from the primordia, the FM and FP stages were collected in Huangfang Town, Jianning County, Fujian Province. As shown in Figure 1, primordia samples were taken before the mushroom caps expanded. FM samples were collected when the white or pale-yellow color of the cap edges had largely disappeared, and the caps were about to emit spores. The FP stage samples were obtained when the caps no longer increased in size, the edges thickened, and spore release had largely ceased. The stipes of the fruiting bodies at the FM and FP stages were removed, and the caps were used for the experiment. Three replicate samples were collected, frozen with liquid nitrogen, and stored at −80 °C. The samples were identified by Professor Chen Tiqiang, and the specimens were stored in the laboratory of traditional Chinese medicine identification, College of Bee Science and Biomedicine, Fujian Agriculture and Forestry University.

### 2.2. Metabolites Analysis

#### 2.2.1. Metabolites Detection

The total triterpenoids from samples at different stages were extracted according to the method described in “Chinese pharmacopoeia 2020” [17]. An amount of 2 g of sample powder was sonicated with 50 mL of ethanol (power: 140 W, frequency: 42 kHz) for 45 min and then filtered and diluted to 100 mL. The extracts were filtered using 0.22 μm filter membrane for analysis. All the measurements were carried out in triplicate.

Samples at different stages were vacuum freeze-dried and pulverized. An amount of 100 mg of the sample powder was dissolved in 1.2 mL of 70% methanol extraction solution, vortexed, centrifuged, and the supernatant was collected. The supernatant was filtered through a microporous membrane, and the filtrate was used for UPLC-MS/MS analysis. All the measurements were carried out in triplicate. Targeted metabolomics analyses were carried out on the LC/MS system that is composed of Waters Acquity I-Class PLUS ultra-high performance liquid tandem, Waters Xevo G2-XS QT of high-resolution mass spectrometer with a Waters Acquity UPLC HSS T3 column (2.1 × 100 mm, 1.8 μm). The A and B mobile phases in positive and negative ion mode consisted of 0.1% formic acid in water and acetonitrile, respectively.

The primary and secondary mass spectra were acquired using a Waters Xevo G2-XS quadrupole time-of-flight (Q-TOF) mass spectrometer in MSe mode. The optimized operating parameters of the ESI ion source were as follows: capillary voltage, 2000 V in positive ion mode and −1500 V in negative ion mode; cone voltage, 30 V; ion source temperature, 150 °C; desolvent gas temperature, 500 °C; the flow rate of backflush gas, 50 L/h; the flow rate of desolventizing gas, 800 L/h; the energy for low collision was 2 V and the energy range for high collision energy was 10~40 V, and the scanning frequency was 0.2 s for a mass spectrum.

#### 2.2.2. Metabolites Data Analysis

The raw data was collected using Mass Lynx TM 4.2 software (Waters, Milford, MA, USA) and processed using Progenesis QI V2.3 software based on the METLIN database and Biomark’s self-built library. The identified compounds were searched for classification and pathway information in KEGG, HMDB, and lipid maps databases. A *t* test was used to calculate the difference significant *p* value of each compound. The screening criteria were FC > 1, *p* value < 0.05, and VIP > 1.

### 2.3. Transcriptome Analysis

#### 2.3.1. Transcriptome Sequencing

Total RNA was extracted from 0.1 mg of a sample using TriZol reagent (Promega, Madison, WI, USA). RNA quality was measured with NanoDrop 2000 (Thermo Fisher Scientific, Wilmington, DE, USA) and Agilent Bioanalyzer 2100 system (Agilent Technologies, Santa Clara, CA, USA). The cDNA libraries were generated using NEBNext Ultra™ RNA Library Prep Kit (NEB, Rowley, MA, USA) as described in the manufacturer’s recommendations. The cDNA libraries were verified using an Agilent 2100 Bioanalyzer and ABI StepOnePlus Real-time PCR system and sequenced using an Illumina HiSeq 4000 (Illumina Inc., San Diego, CA, USA). All the measurements were performed in triplicate.

#### 2.3.2. Transcriptome Data Analysis

Raw reads were processed using the online bioinformatic pipeline tool BMKCloud (www.biocloud.net), and clean data were processed and obtained by removing adapter sequences, reads containing ploy-N and low-quality reads. The clean reads were mapped to the reference genome of *G. lucidum* G.260125-1 (GenBank: AGAX00000000.1) using Hisat2 V2.2.1 software. Genes were annotated and analyzed using the following databases: Nt (non-redundant nucleotide sequences, NCBI), Nr (non-redundant protein sequences, NCBI), Pfam (Protein family), KOG/COG (Clusters of Orthologous Groups of proteins), and KO (KEGG Ortholog database). The levels of gene expression were estimated based on the fragments per kilobase of gene per million mapped fragments (FPKM). DEGs were identified using edgeR. FDR < 0.01 and fold change ≥2 were set as the thresholds for significantly differential expression.

#### 2.3.3. Validation of Genes by qRT-PCR

The RNA extraction and qRT-PCR methods were consistent with those described in our previous research [16]. The transcript level of the genes was calculated according to the 2-MMCT method, and *glyceraldehyde-3-phosphate dehydrogenase* (*GL-GPD*) gene transcripts of *G. lucidum* were used as an internal control. The primers for qRT-PCR analysis are listed in Table 1.

#### 2.3.4. Correlation Analysis

The Pearson correlation method was used to analyze the relationship between the genes and metabolites. The correlation coefficient (CC) and the *p*-value were calculated, and the thresholds were set at |CC| > 0.80 and CCP < 0.05. Differential metabolites and genes were then classified using K-means clustering within each group, followed by plotting according to the classification.

### 2.4. Statistics Analysis

The data’s mean (*n* = 3, biologically independent replicates) ± standard error was calculated using SPSS version 16.0 (IBM^®^ SPSS^®^ Statistics, versus 19.0). Experimental data was analyzed by one-way analysis of variance (ANOVA) followed by Tukey’s test and two-way ANOVA, and *p* < 0.05 was considered statistically significant.

## 3. Results and Discussion

### 3.1. Targeted Metabolomics Analysis

#### 3.1.1. Metabolomic Profiling of *G. lucidum* at Different Growth Stages

**Figure 2:**
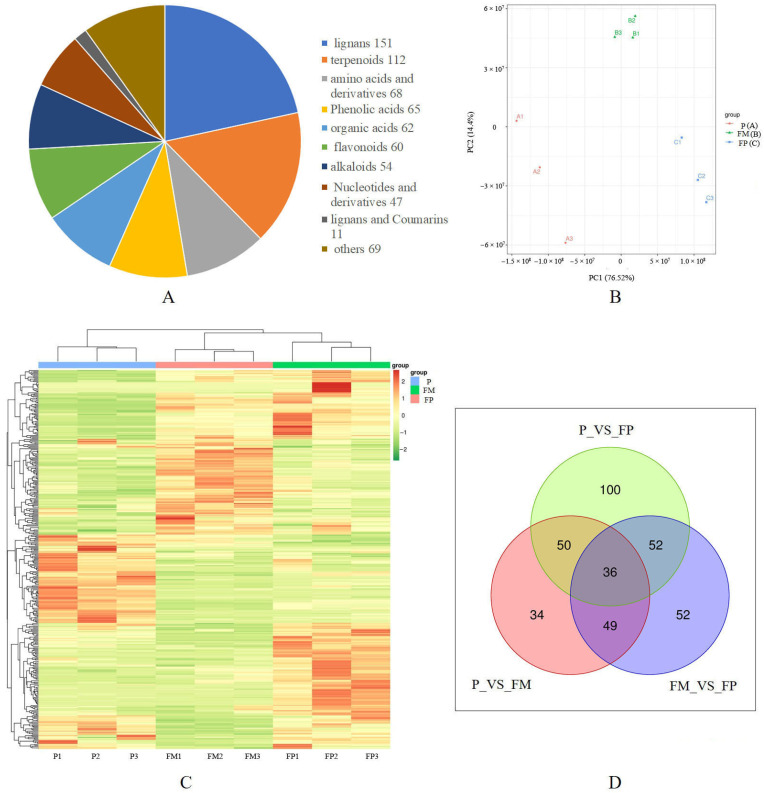
Targeted metabolomics analysis of *G. lucidum* at the different growth stages. (**A**) Total number of metabolites at the different growth stages. (**B**) Principal component analysis (PCA) of metabolomics data. (**C**) The heatmap of metabolic data. (**D**) The number of differential metabolites among groups.

The metabolic changes during the growth stages of *G. lucidum* were investigated using targeted metabolomics approaches. The total content of GTs were 17.035 mg/g, 15.3281 mg/g, and 8.662 mg/g in the primordia, FM, and FP stages, respectively. After producing spores, the total content of GTs in the fruiting body was significantly decreased compared to that in the primordia and FP stages. Using targeted metabolomics approaches, a total of 699 components were detected, including 151 lignans, 112 terpenoids, 68 amino acids and derivatives, 65 phenolic acids, 62 organic acids, 60 flavonoids, 54 alkaloids, 47 nucleotides and derivatives, 11 lignans and coumarins, and 69 other components (Figure 2A) (Supplementary File S1A). Among these components, lipids and terpenoids were found to be the most abundant. A principal component analysis (PCA) showed that the samples from one stage tend to group together and separate from other stages, indicating good repeatability within each group and differences in the chemical composition among the different stages (Figure 2B). The heatmap of hierarchical clustering further demonstrated the quality and reliability of metabolic data (Figure 2C). The content of some components in *G. lucidum* were significantly different during different growth stages. In the P_vs_FM group, there were 169 differential metabolites (Figure 2D), among which 111 were up-regulated and 58 were down-regulated (Supplementary File S1B). During the formation of the primordia into fruiting bodies, the majority of secondary metabolites, including phenolic acids, flavonoids, and organic acids, were up-regulated. In the FM_vs_FP group, there were 189 differential metabolites (Figure 2D), among which 70 were up-regulated and 119 were down-regulated (Supplementary File S1C). In the P_vs_FP group, there were 14 differential metabolites of triterpenoids, with 6 decreasing and 8 increasing (Supplementary File S1D).

The content and types of GTs varied significantly across different regions and growth stages. Some studies have suggested that the content of total triterpenoids was the highest and the variety of triterpenoids was the greatest in the fruiting body, while GT content was the lowest in the mycelium [18,19,20,21]. In the present study, from the trend in metabolite content changes, it can be observed that the majority of metabolites have higher content in the FM stage than in the FP stage. When the white cap edge of *G. lucidum* disappeared, and spores were produced, the cap continued to grow and thicken. This process required the consumption of nutrients to support spore production and fungal growth, resulting in a decrease in the content of most components. However, compared to the primordia, there are 238 differential metabolites, with 137 showing increased levels and 101 showing decreased levels in the FP stage. The majority of phenolic acids, flavonoids, terpenes, and organic acids increased in content after sporulation, while most lipid compounds decreased. In this study, many lipid compounds were detected in *G. lucidum*, with the most abundant being fatty acids, phosphatidylethanolamine, and hemolytic phosphatidylethanolamine. These three types of components are important parts of the plant cells. Fatty acids, the primary constituents of cell membranes, play a crucial role in membrane synthesis and energy storage [22]. Phosphatidylethanolamine, as a type of compound in glycerophospholipids, acts as a major phospholipid in cell membranes [23]. Phosphatidylcholine is a vital component of biological membranes, and its levels may influence the elongation and differentiation processes of fungal hyphal [24]. These three types of compounds play significant nutritional roles in the fungal growth process. In this study, the contents of most lipid substances were decreased in the FP stage, indicating that lipids were utilized for the growth of the fruiting body and the synthesis of certain substances during the spore production process.

#### 3.1.2. GTs Analysis in *G. lucidum* at Different Growth Stages

Triterpenoids are one of the most important bioactive components in *G. lucidum.* In the present study, a total of 67 triterpenoid compounds were detected, including ganoderic acid, ganoderenic acid, lucidenic acid, ganodermanondiol, and ganoderone, among others (Supplementary File S1A). Among these triterpenoids, ganoderic acid has the highest count (37), followed by lucidenic acid (11). A previous study has identified 9384 metabolites in fruiting bodies of *G. lucidum* using non-targeted metabolomics, including polysaccharides, nucleosides, peptides, triterpenoids, and alkaloids. In that study, 36 and 40 triterpenoids were detected in the mycelium and fruiting bodies, respectively [21]. In this study, 67 triterpenoid compounds were detected in the fruiting body of *G. lucidum*. Targeted metabolomics aims to quantitatively or qualitatively measure specific metabolites or metabolic pathways to gain deeper insight into their changes and functions within an organism. Therefore, the use of targeted metabolomics properly can be helpful for detecting a greater number of GTs [25].

In the P_vs_FM group, there were eight differential metabolites of triterpenoids, with three decreasing, including lucidenic acid B, ganoderic acid J, and ganoderenic acid E, and five increasing, including methyl ganoderic acid J, ganoderenic acid H, ganoderenic acid K, ganoderic acid γ, and lucidenic acid P (Supplementary File S1B). In the FM_vs_FP group, there were thirteen differential metabolites of triterpenoids, with six decreasing, including methyl ganoderic acid J, ganoderic acid γ, lucidenic acid P, ganoderic acid D methyl ester, ganoderic acid M, and ganoderic acid N, and seven increasing including ganoderic acid S, ganodermanondiol, ganoderic acid DM, lucidenic acid B, ganolucidic acid D, ganoderic acid V, and ganoderic acid Mj (Supplementary File S1C).

The previous study also revealed that there were certain differences in the composition and types of GTs at different stages, with the lowest quantity and content found in the aerial mycelia. Only one type of triterpenoids was detected in the mycelium of *G. lucidum* culturing on glass paper placed in a PDA solid medium, while 33 and 36 triterpenoids substances were detected in the fermented liquid and mycelium of *G. lucidum*, respectively [16,21]. The content of most ganoderic acids in both the stipe and the cap gradually decreased with the development of *G*. *lucidum* [26]. The total content of triterpenoids significantly decreased after sporulation, suggesting that the sporulation process also affected the content of secondary metabolism. However, the composition and content of GTs were influenced by the cultivation environment and practices, thus the content of individual triterpenoids was also affected [1,21]. In this study, the content of ganolucidic acid D was very low in all three stages; there were high levels of ganoderenic acid E, ganoderenic acid A, ganoderenic acid K, ganoderic acid A, ganoderic acid F, and ganoderic acid Xi in these three stages. Furthermore, ganolucidic acid D, ganoderenic acid K, and ganoderic acid Xi were not detected in previous untargeted metabolomics studies. There was a high content of ganoderiol F in the primordia and FM stage, but it was significantly decreased after spore production. By contrast, ganolucidic acid D and ganoderic acid AP2 were almost undetectable in the primordia stage and the FM stage but increased after spore production. Ganoderic acid Mj was relatively high in the primordia but present only in trace amounts in the fruiting body.

During the cultivation process, excess primordia are removed, leaving only one well-developed primordium. After the spore production stage is completed, the spores and the fruiting bodies (FP stage) are harvested. As a result, a large amount of the primordia and the fruiting bodies (FP stage) that cannot be used as medicinal materials are generated throughout the cultivation process [22]. This study analyzed the chemical composition of *G. lucidum* at different stages and found that some triterpene components were found in relatively high concentrations in the primordia, such as ganoderenic acid E, ganoderic acid F, and ganoderic acid Theta. Some components were found in higher concentrations in the FP stage than in the other stages, including lucidenic acid F, ganoderic acid Theta, ganoderic acid F, ganoderenic acid K, ganoderenic acid H, and ganoderenic acid E. Therefore, new uses for the primordia and the post-spore fruiting bodies can be developed to fully utilize these resources.

#### 3.1.3. Other Plant Components Analysis of *G. lucidum*

Some flavonoids and phenolic acid compounds that are commonly found in plants were detected in *G. lucidum*, such as chlorogenic acid, cryptochlorogenic acid, 6-hydroxymusizin-5-glucoside, naringenin-3-O-rutinoside, and apigenin-7-O-glucoside (Supplementary File S1A) [27]. These constituents are widely distributed in plants but have not been extensively reported in *G. lucidum*. For example, chlorogenic acid is widely distributed in herbal medicines, such as the flower buds of *Lonicera japonica* and *L. macranthoides* [28,29]. Chlorogenic acid possesses various biological activities, such as scavenging free radicals, antibacterial, antiviral, and anti-tumor effects, as well as prevention of diabetes, hyperlipidemia, and hepatitis [30,31]. Currently, chlorogenic acid is widely used in pharmaceuticals, but extracting it from plants is challenging due to issues such as its low content, the complex extraction processes, and the high costs of extraction.

Since endophytic fungi can produce active compounds similar to those of the host, utilizing plant endophytes to obtain active compounds is an important approach. A previous study has reported that a novel fungal NRPS–PKS hybrid enzyme, FnsA, was identified from a plant endophytic fungus *Pestalotiopsis fici* based on genome analysis. This enzyme catalyzes the synthesis of naringenin using cinnamic acid or p-hydroxybenzoic acid as substrates, providing a new pathway for the efficient microbial production of flavonoid compounds [32]. *G. lucidum* is a white rot fungus that grows on plants. There are still few reports on the generation of the same components as the plants in white rot fungi. In addition, the synthesis pathway and regulation of chlorogenic acid in plants have been comprehensively studied. However, there have been no reports of chlorogenic acid synthesis in *G. lucidum*, and research on the function of the key genes related to chlorogenic acid analysis in fungi is also limited. Phenylalanine ammonia-lyase is the first key enzyme in the phenylpropanoid pathway and is involved in the synthesis of many bioactive metabolites, including flavonoids, anthocyanins, and chlorogenic acid [33]. However, the number of introns and conservative sites in fungi are different from those in plants [34]. The enzyme 4-Coumarate coenzyme A ligase (4CL) is a key rate-limiting enzyme in the phenylpropanoid metabolism pathway. This enzyme regulates the synthesis of flavonoids, anthocyanins, and lignin. The interference of *4CL* (Gl21040) in *G. lucidum* resulted in the reduction of flavonoid and lignin content. However, its overexpression increased the content of both flavonoids and lignin [35]. Further research is needed to determine whether the chlorogenic acid synthesis pathway and the functions of related enzymes in fungi are consistent with those in plants. Chlorogenic acid has been found not only in the primordium and fruiting bodies of *G. lucidum* but in the fermentation broth and fermentation mycelium. In addition, there were other flavonoid components with relatively high content in *G. lucidum*, such as 6-HydroxyLuteolin 5-glucoside, Apigenin-7-O-(6′-p-Coumaryl) glucoside, and Apigenin-7-O-neohesperidoside (Rhoifolin). Therefore, *G. lucidum* can be considered as a potential engineering strain for the biosynthesis of phenolic acids and flavonoids.

### 3.2. Transcriptome Analysis in G. lucidum at Different Growth Stages

#### 3.2.1. Transcriptomic Data

**Figure 3:**
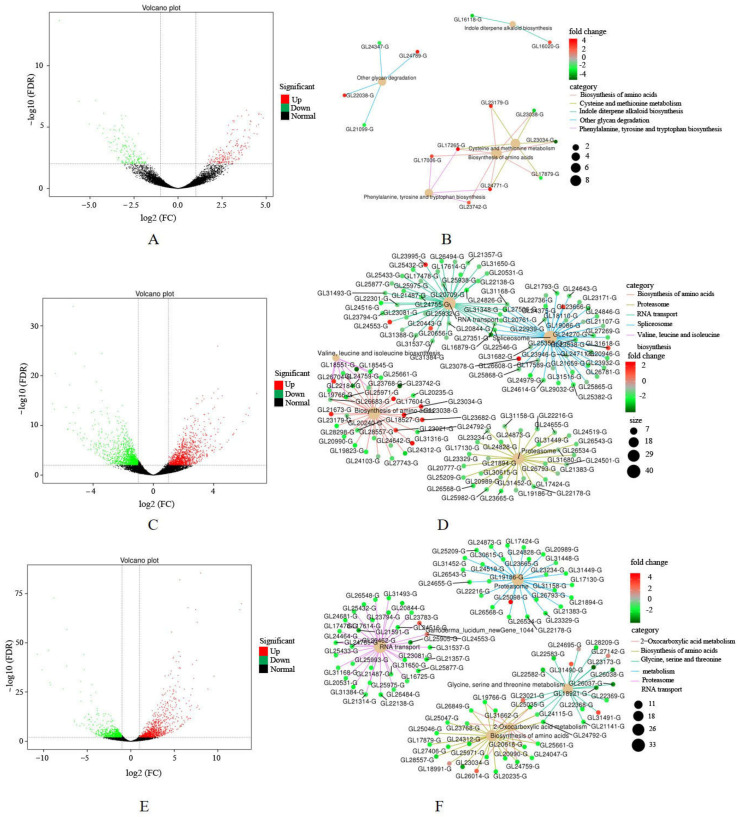
The results of transcriptome analysis of *G. lucidum* at the different growth stages: (**A**) Volcano plot of differential expression of the P_vs_FM group; (**B**) KEGG enrichment network diagram of DEGs of the P_vs_FM group; (**C**) Volcano plot of differential expression of the FM_vs_FP group; (**D**) KEGG enrichment network diagram of DEGs of the FM_vs_FP group; (**E**) Volcano plot of differential expression of the FM_vs_FP group; (**F**) KEGG enrichment network diagram of DEGs of the FM_vs_FP group.

To evaluate the gene expression profile in *G. lucidum*, samples of three stages were used for transcriptome analysis and a total of 18 GB of raw data was obtained. The raw data were deposited in the China National Center for Bioinformation (https://www.cncb.ac.cn/) with accession code CRA021347. There were 371 DEGs in the P_vs_FM group, including 171 down-regulated genes and 200 up-regulated genes (Figure 3A) (Supplementary File S2A). A KEGG enrichment analysis showed that DEGs were mainly classified into biosynthesis of amino acids, tryptophan metabolism, cysteine, and methionine metabolism (Figure 3B). In the FM_vs_FP group, 2567 DEGs were identified, with 1278 down-regulated genes and 1289 up-regulated genes (Figure 3C) (Supplementary File S2B). A KEGG enrichment analysis showed that the DEGs were mainly classified into 2-Oxocarboxylic acid metabolism, biosynthesis of amino acids, and proteasome (Figure 3D).

In the P_vs_FP group, 1941 DEGs were identified, with 862 down-regulated genes and 1079 up-regulated genes (Figure 3E) (Supplementary File S2C). A KEGG enrichment analysis showed that DEGs were mainly classified into biosynthesis of amino acids, proteasome, and RNA transport (Figure 3F). After the primordia are formed, they undergo processes such as stalk differentiation and growth, cap differentiation and growth, fruiting body formation, and sporulation. During sporulation, the cap also thickens simultaneously. The number of DEGs in the P_vs_FM group is much fewer than those in the FM_vs_FP and P_vs_FP groups, indicating that there were more genes involved in the biological regulation processes of spore generation. At the same time, many genes were involved in the growth of *G. lucidum* during this process.

#### 3.2.2. Laccases Associated with the Growth Process

*G. lucidum* is white-rot fungi, which grows saprophytically on trees or wood, utilizing lignin degradation to obtain necessary nutrients for growth. During the growth process of *G. lucidum*, the contents of lignin, cellulose, and hemicellulose in the cultivation substrate continuously decrease [36]. Laccase is a type of polyphenol oxidase that catalyzes the oxidation of various aromatic substrates, playing a significant role in lignin degradation [37].

Additionally, laccase serves important physiological functions during the growth and development of higher fungi, such as fruiting body development, melanin synthesis, and lignin degradation. Currently, some laccases of *G. lucidum* have been cloned, and studies have shown that the expression levels of laccases vary at different growth stages. The laccase of *G. lucidum* has demonstrated potential in the lignocellulosic biomass conversion bioethanol production industry [38,39]. However, no research has yet indicated how laccases are involved in the growth process of *G. lucidum* [40,41]. Therefore, further studies are needed to explore the molecular mechanisms of laccase affecting the morphological development of *G. lucidum*. In the present study, the expression levels of two laccases (GL16401 and GL19134) were higher in the FM and FP than in the primordium, suggesting that these two laccases may play crucial roles in the formation of fruiting bodies. These two laccase genes can be further studied for their roles in the growth and development of *G. lucidum*.

#### 3.2.3. Expression Profile of MVA Pathway Genes

**Figure 4:**
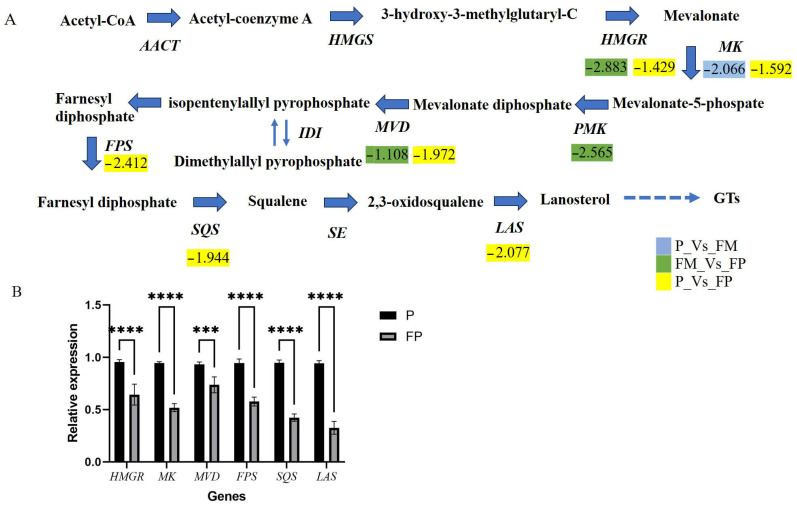
The mevalonate (MVA) pathway of *G. lucidum*: (**A**) The differentially expressed genes involved in the MVA pathway; (**B**) Validation of the relative expression level of genes by qRT-PCR analysis. **** represents extremely significant difference between P and FP, *p* value < 0.0001.

The biosynthesis of GTs is achieved through the MVA pathway. In the MVA pathway, acetyl-CoA (acetyl-coenzyme A, CoA) is converted by 3-hydroxy-3-methyl glutaryl-CoA synthase (HMGS), 3-hydroxy-3-methyl-glutaryl-CoA reductase (HMGR), mevalonate kinase (MVK), phosphomevalonate kinase (PMK), mevalonate pyrophosphate decarboxylase (MVD), isopentenyl diphosphate isomerase (IDI), farnesyl pyrophosphate synthase (FPS), squalene synthase (SQS), and squalene epoxidase (SE) to generate 2,3-oxidosqualene. Subsequently, LAS catalyzes the conversion of 2,3-oxidosqualene into lanosterol. Finally, lanosterol is further modified by CYP450s and glycosyltransferases to produce various structures of GTs. The expression levels of key genes in the MVA pathway can affect the content of lanosterol, thereby influencing the content of triterpenoids. These key enzyme genes have been cloned and identified in *G. lucidum*, and their overexpression could increase the total content of GTs [42,43,44,45,46]. Moreover, they were regulated by methyl jasmonate (MeJA). During the fermentation process, MeJA could significantly induce the production of GTs, and enhance the expression of genes such as *Hmgr*, *Mvd*, *Fps*, *Sqs*, and *Osc* in the biosynthesis pathway. MeJA has been confirmed to regulate triterpenoid synthesis via the ROS signaling pathway [47]. In this study, the expression levels of *HMGR* (GL24088), *MK* (GL17879), *PMK* (GL17808), *MVD* (GL25304), *FPS* (GL25499), *SQS* (GL21690), and *LAS* (GL18675) were all down-regulated in the process of transformation from the primordium stage to the FP stage (Figure 4A). However, from the primordium stage to the FM stage, only the expression level of *MK* was down-regulated. After spore production, the triterpene content decreased, and the expression of these genes was also down-regulated. The TF *Glmhr* could increase the content of lanosterol by regulating the expression level of *FPS* gene [16]. Since the content of lanosterol is closely related to the content of GTs, the synthesis of triterpenoids can be promoted by regulating the expression of key enzymes in the MVA pathway.

The trends in the expression levels of these genes were consistent with the changes in total triterpene content. Six genes involved in the MVA pathway were selected for qRT-PCR analysis. The results of qRT-PCR were nearly consistent with those obtained using the RNA-Seq method, as shown in Figure 4B, demonstrating the reliability of the transcriptome data.

#### 3.2.4. The TFs Related to GTs Synthesis in *G. lucidum*

In this study, a total of 138 differential transcription factors (DTFs) were identified. From the primordium stage to the FM stage, there were 15 DTFs including 9 down-regulated genes and 6 up-regulated genes (Supplementary File S3A). From the primordium stage to the FP stage, there were 51 DTFs, with 34 down-regulated and 9 up-regulated genes (Supplementary File S3B). From the FM stage to the FP stage, there were 101 DTFs, with 79 down-regulated and 22 up-regulated genes (Supplementary File S3C). These DTFs exhibited varying expression levels at different stages. For example, the relative expression levels of *GlC2H2* (GL21684), *GlSET* (GL22435), and *GlJumonji* (GL24195) were the highest in the primordium. On the other hand, *GlC2H2* (GL28755) had the lowest expression level in the primordium. *GlHTH* (GL24216) and *GlGCN5* (GL30332) showed the highest relative expression levels in the FM stage, while *GlMADs* (GL23559), *GlFungal* (GL24393), *GlFungal* (GL28441), and *GlTFII* (GL30164) had the lowest relative expression levels in the same stage. This suggested that these TFs play different roles during various growth stages of *G. lucidum*. Among these 138 DTFs, 27 have been confirmed to be inducible by MeJA. Our previous study showed that the relative expression level of *GlMADS* (GL23559) was positively correlated with the content of GTs induced by MeJA [16]. The MADS-box TFs are highly conserved in fungi, and the most common fungal MADS family TFs are *Mcm1* and *Rlm1*. *GlMADS* (GL23559) was identified as a MADS-box TF homologous to TF *Mcm1* that was involved in hyphal growth, fruiting body formation, secondary metabolism, and virulence [48,49].

GTs are one of the main bioactive components of *G. lucidum.* Therefore, many methods have been used to increase their content. As an important regulatory factor in biological processes, TFs have been reported to play a regulatory role in the biosynthesis of GTs. Overexpression or silencing of TFs could identify whether they play a positive or negative regulatory role in the synthesis of GTs [10,11,12,13,14], but it could not determine what the target genes of these TFs were. Yeast one-hybrid or yeast two-hybrid methods can be used to identify TFs that bind to the gene promoters [50]. Using this approach, the candidate TF *Glmhr* that regulates the *FPS* gene was screened. Additionally, DNA affinity purification and high-throughput sequencing can be used to identify the binding sequences and target sequences of TFs. This method identified the most suitable binding sequence for *SREBP* to be 5′-GRVGRVGRVGR-3′ and identified key target genes involved in the biosynthesis of GTs, ergosterol, and lipids [51]. Using metabolomics and transcriptomics to screen for target genes and employing biotechnological methods to validate the functions of candidate TFs could provide a foundation for the synthetic biology of GTs [52].

#### 3.2.5. CYP450s Related to GTs Synthesis in *G. lucidum*

CYP450s are a superfamily of heme-thiolate monooxygenases that can participate in the biotransformation of steroids and alkane molecules, and the degradation of organic pollutants [53,54]. CYP450 catalysts have a broad range of activity and are capable of catalyzing a variety of different types of oxidative reactions, including hydroxylation, epoxidation, C-C bond oxidation coupling and cleavage. In this study, a total of 93 differentially expressed CYP450 genes were identified. From the primordium stage to the FM stage, there were 17 differentially expressed CYP450 genes including 15 down-regulated genes and 2 up-regulated genes (Supplementary File S4A). From the FM stage to the FP stage, there were 64 differentially expressed CYP450 genes, with 16 down-regulated and 48 up-regulated genes (Supplementary File S4B). From the primordium stage to the FP stage, there were 41 differentially expressed CYP450 genes, with 19 down-regulated and 22 up-regulated genes (Supplementary File S4C).

In the *G. lucidum* genome, 219 potential CYP450 genes were identified, with 197 of them confirmed as functional genes through sequence analysis. Among them, the expression of 78 CYP450 genes was positively correlated with changes in the content of GTs and was consistent with the expression changes in the lanosterol synthase gene, indicating that these 78 CYP450 genes may be related to the biosynthesis of GTs [6]. Subsequently, *CYP5150L8* (GL24883) and *CYP512U6* (GL31761) were confirmed to be involved in the synthesis of GTs. In yeast, the overexpression of *CYP5150L8* can produce HLDOA, with a yield of 14.5 mg/L of HLDOA after 120 h of fermentation. In vitro enzyme assays indicate that *CYP5150L8* can catalyze a three-step sequential oxidation at the C-26 position of lanosterol, ultimately generating HLDOA [17]. In vitro experiments have demonstrated that *CYP512U6* can hydroxylate the C-23 position of ganoderic acid DM and TR, resulting in the production of hainanic acid A and ganoderic acid Jc, respectively [55]. In this study, it was found that the expression level of *CYP512U6* was down-regulated during the fruiting body stage (including the FM and FP stages). The expression level of *CYP5150L8* decreased from the primordium stage to the FM stage, as well as from the FM stage to the FP stage. This trend is consistent with the changes in the total GTs content of *G. lucidum.*

Among the differential P450 genes, the CYP5150 and CYP5139 families have the largest numbers, with 18 and 17 members, respectively. In fungal genomes, the large number of genes in these two families can be attributed to the dramatic changes caused by gene duplication [56,57]. Some CYP450 genes in these two families exhibit catalytic activity. Except for CYP5150L8, the purified CYP5150A2 was able to hydroxylate 4-propylbenzoic acid with NADPH-dependent cytochrome P450 oxidoreductase as the single redox partner [58]. CYP5150AP3 from *Thanatephorus cucumeris* could catalyze the 7β-hydroxylation of 11-deoxycortisol, as well as the 6β- and 7β-hydroxylation of testosterone. Meanwhile, CYP5150AN1 could catalyze the 2β-hydroxylation of 11-deoxycortisol [59]. In this study, the screening of differential CYP450 genes provided data for the discovery of additional functional genes.

### 3.3. Combined Transcriptome and GTs Analysis

To gain more insights into the regulation mechanism of the biosynthesis of GTs, the correlation between DEGs and the differential metabolites was evaluated using Pearson’s correlation analysis.

**Figure 5:**
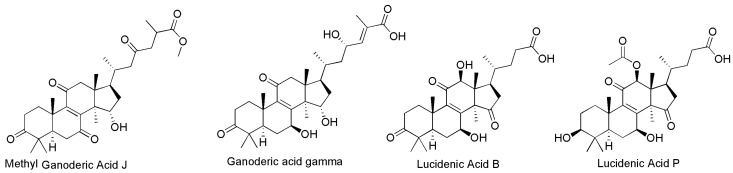
The molecular structures of the triterpenes.

Six TFs and two CYP450s were significantly associated with four GTs metabolites, including methyl ganoderic Acid J, ganoderic acid γ, lucidenic acid P, and lucidenic acid B (Figure 5). *GlMADs* (GL23559) and *GlHTH* (GL24216) were positively correlated with methyl ganoderic acid J, while *GlFungal* (GL24393), *GlFungal* (GL28441), and *CYP5035M1* (GL21420) showed a negative correlation with methyl ganoderic acid J. TF *GlMADs* (GL23559) showed a positive correlation with ganoderic acid γ, while *GlHTH* (GL24216), *GlFungal* (GL24393), *CYP5035M1* (GL21420), and *CYP5359Y2* (GL17412) showed a negative correlation with ganoderic acid Gamma. TF *GlMADs* (GL23559) was positively correlated with lucidenic acid P, while *CYP5035M1* (GL21420) and *CYP5359Y2* (GL17412) were negatively correlated with lucidenic acid P. TFs *GlFungal* (GL24393), *GlFungal* (GL28441), *GlTFII* (GL30164), and *CYP5359Y2* (GL17412) were positively correlated with lucidenic acid B, while *GlGCN5* (GL30332) was negatively correlated with lucidenic acid B.

The results showed that *GlMADs* (GL23559) played positive roles in the biosynthesis of methyl ganoderic acid J, ganoderic acid gamma, and lucidenic acid P, while *CYP5035M1* (GL21420) played a positive role in the biosynthesis of methyl ganoderic acid J, ganoderic acid Gamma, and lucidenic acid P, suggesting that they may regulate the biosynthesis of different triterpenoids components as activators or inhibitors. Through co-expression analysis, we identified TFs and CYP450s related to the synthesis of some GTs components. However, how these TFs are involved in the synthesis of these components still requires further research.

## 4. Conclusions

In the current study, targeted metabolomics and transcriptomics analysis were used to screen for the genes that are involved in the biosynthetic pathway of GTs. Three stages of *G. lucidum*, including the primordia, FM and FP, were selected for this study. As a result, a total of 699 components were detected, mainly including lignans, terpenoids, amino acids and derivatives, and phenolic acids. In the P_vs_FM group, there were eight differential metabolites of triterpenoids, with three decreasing and five increasing. The comparison between the FM stage and the FP stage revealed that there were 13 differential metabolites of triterpenoids; the transcriptomics analysis showed that there were 371 DEGs in the P_vs_FM group and 2567 DEGs in the FM_vs_FP group. A correlation analysis revealed that six TFs and two CYP450s were significantly associated with four triterpenoid components, indicating these genes were closely related to the synthesis of these GTs components. This is the first study to report the use of targeted metabolomics and transcriptomics to analyze the differential metabolites and genes across three growth stages. This study provides valuable information for investigating the regulation of triterpene biosynthesis and related genes at different growth stages of *G. lucidum*.

## Figures and Tables

**Table 1:** Primers sequences for qRT-PCR.

Primers	Sequences(5′→3′)
*GPD*-F	CTCCTTCACGGAGACATT
*GPD*-R	TAACACCCGCAGACGAACA
GL18675-*las*-F	CTTCCGCAAGCACTACCCG
GL18675-*las*-R	AGCAGATGCCCCACGAGCC
GL24922-*hmgs*-F	ACACGACGGAGATTCAGGAG
GL24922-*hmgs*-R	GATGTACTTGCGGCGGTATT
GL24088-*hmgr*-F	GCGTCGGTAACATGATCCTT
GL24088-*hmgr*-R	GACAAGACTCCGCGAATAG
GL22068-*fps*-F	GGTTGGTGTGTCGAGTTCCT
GL22068-*fps*-R	TGACGATCTTTTGGTGCTTG
GL17879-*mk*-F	TTCGCTGTTACGGTCTTA
GL17879-*mk*-R	GTGGTTGTAGTTGCTGATAT
GL25304-*mvd*-F	GCATTAAGGAGATGAAGG
GL25304-*mvd*-R	GAGATGTGAACGGAGTAG
GL21690-*sqs*-F	CTTATTCTACCTGGTGCTA
GL21690-*sqs*-R	TGGAATTGTCGGAGTATG

## Data Availability

The datasets presented in this study had been submitted to “China National Center for Bioinformation (Beijing Institute of Genomics, Chinese Academy of Sciences)”, https://www.cncb.ac.cn/, accession code: CRA021347.

## Supplementary Materials

The following supporting information can be downloaded at: https://www.mdpi.com/article/10.3390/jof11010057/s1. Supplementary File S1. Targeted metabonomic analysis of *G. lucidum* at the different growth stages. Supplementary File S1A. All metabonomic profiling of *G. lucidum* at the different growth stages. Supplementary File S1B. Differential metabolites of GTs between P stage and FM stage. Supplementary File S1C. Differential metabolites of GTs between FM stage and FP stage. Supplementary File S1D. Differential metabolites of GTs between P stage and FP stage. Supplementary File S2. Differentially expressed genes (DEGs) of *G. lucidum* at the different growth stages. Supplementary File S2A. DEGs between primordia stage and FM stage. Supplementary File S2B. DEGs between FM stage and FP stage. Supplementary File S2C. DEGs between P stage and FP stage. Supplementary File S3. Differentially expressed transcription factors (DETs) of *G. lucidum* at the different growth stages Supplementary File S3A. DETs between primordia stage and FM stage. File S3B. DETs between FF stage and FP stage. Supplementary File S3C. DETs between P stage and FP stage. Supplementary File S4. Differentially expressed CYP450s of *G. lucidum* at the different growth stages. Supplementary File S4A. Differentially expressed CYP450s between P stage and FM stage. Supplementary File S4B. Differentially expressed CYP450s between FM stage and FP stage. Supplementary File S4C. Differentially expressed CYP450s between P stage and FP stage.

## References

[1] Wang L., Li J.Q., Zhang J., Li Z.M., Liu H.G., Wang Y.Z. (2020). Traditional uses, chemical components and pharmacological activities of the genus Ganoderma P. Karst.: A review. RSC Adv..

[2] Sułkowska-Ziaja K., Balik M., Szczepkowski A., Trepa M., Zengin G., Kała K., Muszyńska B. (2023). A Review of Chemical Composition and Bioactivity Studies of the Most Promising Species of *Ganoderma* spp.. Diversity.

[3] Hu Z., Du R., Xiu L., Bian Z., Ma C., Sato N., Hattori M., Zhang H., Liang Y., Yu S. (2020). Protective effect of triterpenes of *Ganoderma lucidum* on lipopolysaccharide-induced inflammatory responses and acute liver injury. Cytokine.

[4] Ryu D.H., Cho J.Y., Sadiq N.B., Kim J.C., Lee B., Hamayun M., Lee T.S., Kim H.S., Park S.H., Nho C.W. (2021). Optimization of antioxidant, anti-diabetic, and anti-inflammatory activities and ganoderic acid content of differentially dried *Ganoderma lucidum* using response surface methodology. Food Chem..

[5] Zeng P., Guo Z., Zeng X., Hao C., Zhang Y., Zhang M., Liu Y., Li H., Li J., Zhang L. (2018). Chemical, biochemical, preclinical and clinical studies of *Ganoderma lucidum* polysaccharide as an approved drug for treating myopathy and other diseases in China. J. Cell. Mol. Med..

[6] Chen S., Xu J., Liu C., Zhu Y., Nelson D.R., Zhou S., Li C., Wang L., Guo X., Sun Y. (2012). Genome sequence of the model medicinal mushroom *Ganoderma lucidum*. Nat. Commun..

[7] Wang W.F., Xiao H., Zhong J.J. (2018). Biosynthesis of a ganoderic acid in Saccharomyces cerevisiae by expressing a cytochrome P450 gene from *Ganoderma lucidum*. Biotechnol. Bioeng..

[8] Yuan W., Jiang C., Wang Q., Fang Y., Wang J., Wang M., Xiao H. (2022). Biosynthesis of mush-room-derived type II ganoderic acids by engineered yeast. Nat. Commun..

[9] Afrin S., Huang J.J., Luo Z.Y. (2015). JA-mediated transcriptional regulation of secondary metabolism in medicinal plants. Sci. Bull..

[10] Wu F.L., Zhang G., Ren A., Dang Z.H., Shi L., Jiang A.L., Zhao M.W. (2016). The pH-responsive transcription factor PacC regulates mycelial growth, fruiting body development, and ganoderic acid biosynthesis in *Ganoderma lucidum*. Mycologia.

[11] Wang S., Shi L., Hu Y., Liu R., Ren A., Zhu J., Zhao M. (2018). Roles of the Skn7 response regulator in stress resistance, cell wall integrity and GA biosynthesis in *Ganoderma lucidum*. Fungal Genet. Biol..

[12] Zhang G., Ren A., Shi L., Zhu J., Jiang A., Shi D., Zhao M. (2018). Functional analysis of an APSES transcription factor (GlSwi6) involved in fungal growth, fruiting body development and ganoderic-acid biosynthesis in *Ganoderma lucidum*. Microbiol. Res..

[13] Hu Y., Lian L., Xia J., Hu S., Xu W., Zhu J., Ren A., Shi L., Zhao M.W. (2020). Influence of PacC on the environmental stress adaptability and cell wall components of *Ganoderma lucidum*. Microbiol. Res..

[14] Liang H., Zhong J.J. (2020). Role of calcineurin-responsive transcription factor CRZ1 in ganoderic acid biosynthesis by *Ganoderma lucidum*. Process Biochem..

[15] Wang Z., Chen J., Ding J., Han J., Shi L. (2023). GlMPC activated by GCN4 regulates secondary metabolism under nitrogen limitation conditions in *Ganoderma lucidum*. mBio.

[16] Xu X., Zhu F., Zhu Y., Li Y., Zhou H., Chen S., Ruan J. (2022). Transcriptome profiling of transcription factors in *Ganoderma lucidum* in response to methyl jasmonate. Front. Microbiol..

[17] National Pharmacopoeia Committee (2020). Pharmacopoeia of People′s Republic of China, Part 1.

[18] Liu J., Kenji K., Atsuko F., Shuhei K., Yoshitaro S., Kuniyoshi S., Ryuichiro K. (2012). Quantitative determination of the representative triterpenoids in the extracts of *Ganoderma lucidum* with different growth stages using high-performance liquid chromatography for evaluation of their 5α-reductase inhibitory properties. Food Chem..

[19] Wang C., Liu X., Lian C., Ke J., Liu J. (2019). Triterpenes and Aromatic Meroterpenoids with Antioxidant Activity and Neuroprotective Effects from *Ganoderma lucidum*. Molecules.

[20] Yang Y.L., Zhang H., Zuo J.H., Gong X., Yi F., Zhu W., Li L. (2019). Advances in research on the active constituents and physiological effects of *Ganoderma lucidum*. Biomed. Dermatol..

[21] Xie C., Yan S., Zhang Z., Gǒng W., Zhu Z., Zhou Y., Li Y., Hu Z., Ai L., Peng Y. (2020). Mapping the metabolic signatures of fermentation broth, mycelium, fruiting body and spores powder from *Ganoderma lucidum* by untargeted metabolomics. LWT.

[22] de Carvalho C.C.C.R., Caramujo M.J. (2018). The Various Roles of Fatty Acids. Molecules.

[23] Cassilly C.D., Reynolds T.B. (2018). PS, It’s Complicated: The Roles of Phosphatidylserine and Phosphatidylethanolamine in the Pathogenesis of *Candida albicans* and other Microbial Pathogens. J. Fungi.

[24] Suzawa T., Iwama R., Fukuda R., Horiuchi H. (2024). Phosphatidylcholine levels regulate hyphal elongation and differentiation in the filamentous fungus *Aspergillus oryzae*. Sci. Rep..

[25] Ribbenstedt A., Ziarrusta H., Benskin J.P. (2018). Development, characterization and comparisons of targeted and non-targeted metabolomics methods. PLoS ONE.

[26] Xia J., He X., Yang W., Song H., Yang J., Zhang G., Yang Z., Chen H., Liang Z., Kollie L. (2024). Unveiling the distribution of chemical constituents at different body parts and maturity stages of Ganoderma lingzhi by combining metabolomics with desorption electrospray ionization mass spectrometry imaging (DESI). Food Chem..

[27] Hu S., Zhao R., Chi X., Chen T., Li Y., Xu Y., Zhu B., Hu J. (2024). Unleashing the power of chlorogenic acid: Exploring its potential in nutrition delivery and the food industry. Food Funct..

[28] Yang C., Zhang N., Wu S., Jiang C., Xie L., Yang F., Yu Z. (2023). A Comparative Analysis of the Chloroplast Genomes of Three Lonicera Medicinal Plants. Genes.

[29] Feng Y., Zhang G., Zhu P., Zhu W., Li Y., Fan X.W. (2023). Metabolite profiles and antibacterial and antioxidant activities of leaf extracts of five Lonicera species: A comparative study. Chem. Biol. Technol. Agric..

[30] Onakpoya I.J., Spencer E.A., Thompson M.J., Heneghan C.J. (2015). The effect of chlorogenic acid on blood pressure: A systematic review and meta-analysis of randomized clinical trials. J. Hum. Hypertens..

[31] Naveed M., Hejazi V., Abbas M., Kamboh A.A., Khan G.J., Shumzaid M., Ahmad F., Babazadeh D., Fang X., Modarresi-Ghazani F. (2018). Chlorogenic acid (CGA): A pharmacological review and call for further research. Biomed. Pharmacother..

[32] Zhang H., Li Z., Zhou S., Li S.M., Ran H., Song Z., Yu T., Yin W.B. (2022). A fungal NRPS-PKS enzyme catalyses the formation of the flavonoid naringenin. Nat. Commun..

[33] Amjad M., Wang Y., Han S., Haider M.Z., Sami A., Batool A., Shafiq M., Ali Q., Dong J., Sabir I.A. (2024). Manzoor MA. Genome wide identification of phenylalanine ammonia-lyase (PAL) gene family in *Cucumis sativus* (cucumber) against abiotic stress. BMC Genom. Data.

[34] Hyun M.W., Yun Y.H., Kim J.Y., Kim S.H. (2011). Fungal and Plant Phenylalanine Ammonia-lyase. Mycobiology.

[35] Meng L., Zhou R., Liang L., Zang X., Lin J., Wang Q., Wang L., Wang W., Li Z., Ren P. (2024). 4-Coumarate-CoA ligase (4-CL) enhances flavonoid accumulation, lignin synthesis, and fruiting body formation in *Ganoderma lucidum*. Gene.

[36] Zhou S., Zhang X., Ma F., Xie S., Tang C., Tang Q., Zhang J. (2021). Integrative Analysis of Selected Metabolites and the Fungal Transcriptome during the Developmental Cycle of Ganoderma lucidum Strain G0119 Correlates Lignocellulose Degradation with Carbohydrate and Triterpenoid Metabolism. Appl. Environ. Microbiol..

[37] Patel N., Shahane S., Shivam, Majumdar R., Mishra U. (2019). Mode of Action, Properties, Production, and Application of Laccase: A Review. Recent Pat. Biotechnol..

[38] Sitarz A.K., Mikkelsen J.D., Højrup P., Meyer A.S. (2013). Identification of a laccase from Ganoderma lucidum CBS 229.93 having potential for enhancing cellulase catalyzed lignocellulose degradation. Enzyme Microb. Technol..

[39] Fang Z., Liu X., Chen L., Shen Y., Zhang X., Fang W., Wang X., Bao X., Xiao Y. (2015). Identification of a laccase Glac15 from *Ganoderma lucidum* 77002 and its application in bioethanol production. Biotechnol. Biofuels.

[40] Zill-e-Huma A., Shakil A. (2015). *Ganoderma lucidum*: A case study for laccase biosynthesis. J. Phytopathol..

[41] Zhou S., Zhang J., Ma F., Tang C., Tang Q., Zhang X. (2018). Investigation of lignocellulolytic enzymes during different growth phases of *Ganoderma lucidum* strain G0119 using genomic, transcriptomic and secretomic analyses. PLoS ONE.

[42] Shang C.H., Zhu F., Li N., Ou-Yang X., Shi L., Zhao M.W., Li Y.X. (2008). Cloning and characterization of a gene encoding hmg-coa reductase from *Ganoderma lucidum* and its functional identification in yeast. Biosci. Biotechnol. Biochem..

[43] Ding Y.X., Ou-Yang X., Shang C.H., Ren A., Shi L., Li Y.X., Zhao M.W. (2008). Molecular cloning, characterization, and differential expression of a farnesyl-diphosphate synthase gene from the basidiomycetous fungus *Ganoderma lucidum*. Biosci. Biotechnol. Biochem..

[44] Ren A., Qin L., Shi L., Dong X., Mu D.S., Li Y.X., Ming W.Z. (2010). Methyl jasmonate induces ganoderic acid biosynthesis in the basidiomycetous fungus *Ganoderma lucidum*. Bioresour. Technol..

[45] Shi L., Qin L., Xu Y., Ren A., Fang X., Mu D., Tan Q., Zhao M. (2012). Molecular cloning, characterization, and function analysis of a mevalonate pyrophosphate decarboxylase gene from *Ganoderma lucidum*. Mol. Biol. Rep..

[46] Ren A., Ouyang X., Shi L., Jiang A.L., Mu D.S., Li M.J., Han Q., Zhao M.W. (2013). Molecular characterization and expression analysis of glhmgs, a gene encoding hydroxymethylglutaryl-coa synthase from *Ganoderma lucidum* (ling-zhi) in ganoderic acid biosynthesis pathway. World J. Microbiol. Biotechnol..

[47] Shi L., Gong L., Zhang X., Ren A., Gao T., Zhao M. (2015). The regulation of methyl jasmonate on hyphal branching and GA biosynthesis in *Ganoderma lucidum* partly via ROS generated by NADPH oxidase. Fungal Genet. Biol..

[48] Yang C., Liu H., Li G., Liu M., Yun Y., Wang C., Ma Z., Xu J.R. (2015). The MADS-box transcription factor FgMcm1 regulates cell identity and fungal development in Fusarium graminearum. Environ. Microbiol..

[49] Zhao X., Yang X., Lu Z., Wang H., He Z., Zhou G., Luo Z., Zhang Y. (2019). MADS-box transcription factor Mcm1 controls cell cycle, fungal development, cell integrity and virulence in the filamentous insect pathogenic fungus *Beauveria bassiana*. Environ. Microbiol..

[50] Xu X.L., Lai R.C., Chen T.Q., Shi L.C., Chen S.L. (2020). Construction of yeast one-hybrid library and screening of transcription factors regulating FPS expression in Ganoderma lucidum. Chin. Tradit. Herb. Drugs.

[51] Liu Y.N., Wu F.Y., Tian R.Y., Shi Y.X., Xu Z.Q., Liu J.Y., Huang J., Xue F.F., Liu B.Y., Liu G.Q. (2023). The bHLH-zip transcription factor SREBP regulates triterpenoid and lipid metabolisms in the medicinal fungus Ganoderma lingzhi. Commun. Biol..

[52] Meng L., Zhou R.Y., Lin J.L., Zang X.Z., Wang Q.J., Wang P.M., Wang L., Li Z., Wang W. (2022). Transcriptome and metabolome analyses reveal transcription factors regulating ganoderic acid biosynthesis in *Ganoderma lucidum* development. Front. Microbiol..

[53] Christ B., Xu C., Xu M., Li F.S., Wada N., Mitchell A.J., Han X.L., Wen M.L., Fujita M., Weng J.K. (2019). Repeated evolution of cytochrome P450-mediated spiroketal steroid biosynthesis in plants. Nat. Commun..

[54] Lin S., Wei J., Yang B., Zhang M., Zhuo R. (2022). Bioremediation of organic pollutants by white rot fungal cytochrome P450: The role and mechanism of CYP450 in biodegradation. Chemosphere.

[55] Yang C., Li W., Li C., Zhou Z., Xiao Y., Yan X. (2018). Metabolism of ganoderic acids by a Ganoderma lucidum cytochrome P450 and the 3-keto sterol reductase ERG27 from yeast. Phytochemistry.

[56] Syed K., Nelson D.R., Riley R., Yadav J.S. (2013). Genomewide annotation and comparative genomics of cytochrome P450 monooxygenases (P450s) in the polypore species *Bjerkandera adusta*, *Ganoderma* sp. and *Phlebia brevispora*. Mycologia.

[57] Venkatesh M., Jongsun P., Natalie D.F.A., Bongsoo P., Jaeyoung C., Yong H.L., Seogchan K. (2012). Systematic and searchable classification of cytochrome P450 proteins encoded by fungal and oomycete genomes. BMC Genom..

[58] Hirofumi I., Hiroyuki W. (2012). Heterologous expression and mechanistic investigation of a fungal cytochrome P450 (CYP5150A2): Involvement of alternative redox partners. Arch. Biochem. Biophys..

[59] Lu W., Feng J., Chen X., Bao Y.J., Wang Y., Wu Q.Q., Ma Y.H., Zhu D. (2019). Distinct Regioselectivity of Fungal P450 Enzymes for Steroidal Hydroxylation. Appl. Environ. Microbiol..

